# Open questions: Epigenetics and the role of heterochromatin in development

**DOI:** 10.1186/1741-7007-11-21

**Published:** 2013-03-04

**Authors:** Susan M Gasser

**Affiliations:** 1 Friedrich Miescher Institute for Biomedical Research, Maulbeerstrasse 66, 4058 Basel, Switzerland

## 

Over the last 8 years my laboratory has begun to explore the question of epigenetic changes during differentiation and development, and its link to nuclear organization, which indeed changes during cell differentiation. We developed a method to image chromatin domains in the living nematode *Caenorhabditis elegans*, because the worm provides opportunities to study the effects of nuclear organization on gene expression during well-characterized cellular differentiation events. We found that the nuclear localization of tissue-specific promoters, which seem to have no fixed positions in early embryos, shift within the nucleus during cell differentiation, so that they are positioned near the nuclear lamina when they are inactive, and are restricted to the center of the nucleus when expressed. This was true in four different cell types representing distinct cell lineages (muscle, gut, neurons and hypoderm) [1]. The movement to the nuclear center coincided with transcriptional induction, but was not simply a result of transcription: active genes can be tethered to the nuclear envelope and stress-induced promoters, such as the heat-shock activated promoter *hsp-16.2*, actually bind the nuclear pore in their active state [2]. We then identified lysine methylation on histone H3 lysine 9 (H3K9) as the critical signal for anchoring heterochromatin to the nuclear envelope. Not only tri-methylation, but apparently mono- and di-methylation as well, deposited by the sequential action of two histone methyl transferases (HMT) called MET-2 and SET-25, led to the sequestration of the modified chromatin at the nuclear envelope [3]. A second, and very unexpected finding was that *C. elegans *embryos can develop into adult worms with the full range of differentiated tissues, without any histone H3K9 methylation (H3K9me) whatsoever.

## What is the point of heterochromatin?

So, what role does heterochromatin serve during development, and why bother to sequester it at the nuclear envelope? The notion that cell-type differentiation requires heterochromatin, as defined by the conserved mark of histone H3K9 methylation and its ligand HP-1, may need revision. Moreover, the ability of a multicellular organism to survive and differentiate without H3K9 methylation is unlikely to be unique to *C. elegans*, since the relevant HMTs are quite conserved: MET-2 is the homolog of human SetDB1 or ESET, and catalytic domain of SET-25 strongly resembles the human HMT, G9a or hSUV3-9, and H3K9 methylation also correlates with lamin-associated domains in mouse and human cells. Thus, the following questions, which address some fundamental principles of epigenetics in development, face the entire field.

### 1) What is the crosstalk between different types of chromatin mediated silencing?

The surprising finding that worm tissues can be formed in the absence of histone H3K9 methylation raises the question about compensatory pathways. Does Polycomb-mediated silencing compensate for the loss of HP-1/H3K9me type heterochromatin? Can the two pathways silence the same domains, if needed? Are there other pathways of repression that repress inappropriate tissue-specific genes, or are the 'inappropriate' tissue-specific genes simply silent because the right combination of transcription factors are missing?

### 2) Why is inactive chromatin sequestered in clusters around the nucleolus and at the nuclear lamina?

The striking changes in nuclear organization that arise during differentiation, and which distinguish populations of differentiated cells, raise the question of whether subnuclear position plays a role in cell-type memory of gene expression patterns. By identifying the chromatin marks, their ligands, and the anchors for these ligands at the nuclear envelope, it should be possible to generate mutations in the relevant genes and examine closely the phenotypes that result from the loss of perinuclear anchoring. There is clear evidence that the mutation of nuclear lamin A causes degenerative disease in man, and loss of muscle integrity in worms [4]. Therefore, nuclear organization is likely to play a role in tissue homeostasis. But what is that role? Do the disease phenotypes reflect changes in transcription, or changes in genome stability? It will also be important to determine whether the sequestration of chromatin at the nuclear periphery becomes more important when other pathways of control are compromised.

### 3) What signals affect heterochromatin organization in response to the environment?

We are beginning to understand the impact of histone and DNA modifications, and the enzymes that deposit or remove them, on transcription. However, we still know little about what triggers these epigenetic changes. Epigenetic modifications respond to the environment and to extreme nutrient conditions, but what actually triggers the change in histone or DNA methylation? If epigenetic marks are in place to prevent drastic misregulation during stressful periods - then most likely the heterochromatin responds to stress-induced kinases that are activated under environmental stress. Developmental processes themselves may require chromatin-modifying kinase signals. Finding which signaling pathways regulate chromatin modifiers, and how, will be a fruitful area of research.

### 4) Is there an epigenetic code that restricts transcription factor activity, or is the epigenetic landscape merely a result of transcriptional factor regulation?

The finding that sequence elements target DNA methylation in mammalian cells [5] - just as Polycomb response elements (PREs) target Polycomb repression in flies, and silencers target SIR-mediated repression in yeast - leads one to wonder if all transcriptional control is sequence driven, with the epigenetic marks simply playing a modulatory role. To use a metaphor, perhaps the keys and strings of the piano are the transcription factors, while the epigenetic marks are the piano pedals that just dampen or sustain the tones. If this is the case, is the interplay similar in all tissues or does each have its own logic for crosstalk between epigenetic marks and transcription factors? It should be a major goal of epigenetics research to elucidate the general regulatory principles that define how transcription factors respond to specific patterns of epigenetic marks - if they exist, that is. One will only know by comparing different systems of differentiation, some normal and others aberrant, to uncover the universal rules for the interplay. Such rules may well be broken during oncogenesis.

If we are able to answer these four questions, we may start to understand the true nature of epigenetic control.
